# Mikto-Arm Stars as Soft-Patchy Particles: From Building Blocks to Mesoscopic Structures

**DOI:** 10.3390/polym13071114

**Published:** 2021-04-01

**Authors:** Petra Bačová, Dimitris G. Mintis, Eirini Gkolfi, Vagelis Harmandaris

**Affiliations:** 1 Computation-Based Science and Technology Research Center, The Cyprus Institute, 20 Constantinou Kavafi Str., Nicosia 2121, Cyprus; dimitris.g.mintis@gmail.com (D.G.M.); harman@uoc.gr (V.H.); 2 Institute of Applied and Computational Mathematics (IACM), Foundation for Research and Technology Hellas (FORTH), GR-70013 Heraklion, Crete, Greece; eirini.gkolfi@gmail.com; 3 Department of Mathematics and Applied Mathematics, University of Crete, GR-70013 Heraklion, Crete, Greece

**Keywords:** atomistic simulations, mikto-arm stars, patchy particles

## Abstract

We present an atomistic molecular dynamics study of self-assembled mikto-arm stars, which resemble patchy-like particles. By increasing the number of stars in the system, we propose a systematic way of examining the mutual orientation of these fully penetrable patchy-like objects. The individual stars maintain their patchy-like morphology when creating a mesoscopic (macromolecular) self-assembled object of more than three stars. The self-assembly of mikto-arm stars does not lead to a deformation of the stars, and their shape remains spherical. We identified characteristic sub-units in the self-assembled structure, differing by the mutual orientation of the nearest neighbor stars. The current work aims to elucidate the possible arrangements of the realistic, fully penetrable patchy particles in polymer matrix and to serve as a model system for further studies of nanostructured materials or all-polymer nanocomposites using the mikto-arm stars as building blocks.

## 1. Introduction

Polymer nanoparticles, due to their versatility, can serve as ideal candidates for building blocks in the design of pre-defined highly ordered superstructures. Building on the knowledge of the properties of the basic building unit, one can come up with a hierarchical self-assembly approach or a bottom-up strategy to produce from single (nano)particles a complex material with predefined properties. In contrast to the nanofillers which comprise polymer-covered inorganic particle [1,2,3,4,5], the all-polymer nanoparticles are usually soft and adapt their shape similarly to soft colloids [6]. The variety in polymer types and architectures is the key to open the door to creativity, for example, one can tune the composition of the polymer-based nanoparticles [7,8,9,10] to control the mutual interactions or modify their architecture to either form single-chain nanoparticles or particles with branch-like architecture to control their dynamics.

Combining two or more chemically distinct components leads to the formation of nanostructured materials, more specifically, in the case of the particles, to the formation of multi-compartment nanostructured surface-anisotropic particles such as Janus [11,12,13] and patchy ones [14,15]. The Janus particles, which were firstly introduced by Casagrade and co-workers in 1989 [16], consist of two hemispheres (patches) with dissimilar properties (that may differ in physical, chemical and/or electrical properties) [17], whereas patchy particles consist of multiple such discrete surface regions (patches) [18] exhibiting highly directional interactions which can serve as directional fabrication bonding sites for the self-assembly into highly ordered superstructures [19]. The ability of colloidal patchy particles to self-knot has attracted remarkable attention in the potential usage of such soft patchy particles in the bottom-up fabrication of photonic crystals and sensors [20], electronics [21], and targeted drug delivery [22,23,24,25]. Despite the availability of several patchy particle fabrication techniques (e.g., glancing-angle vapor deposition, nanoparticle lithography, particle lithography, micro-fluidics) [26,27,28,29,30,31,32,33,34,35], the surface-anisotropic nature of the patchy particles only enables a certain degree of control over the assembly process. Top-down manufacturing approaches such as photolithography [36,37] do not enable efficient controlling over the assembly process [34], whereas the directed- or self-assembly of patchy particles (either known as the “bottom-up” or hierarchical assembly process) strategy enables better controlling of the interactions between the primary building units (patches) and the arrangement of the number and spatial distribution of patches on the surface [38,39] as well as exploiting better controlling of the position of the kinetic assembly pathways on the single particle building unit level [9,40]. However, scalable methods that will enable the large-scale manufacturing of patchy colloids still remain a challenge and further research needs to be conducted [41].

Molecular simulations have often been regarded as a very useful tool for directly imaging the assembly behavior of patchy particles at the nano-scale level, as they can enable the systematic study of the geometry of the patchy particles as well as address the mechanisms of interactions that arise between the patches [42,43,44,45,46]. Together with recent theoretical studies [47,48], which examined the functional and mechanical properties of exotic structures that have been formed based on a “bottom-up” approach, these works have been paving the way for experimentalists to investigate novel and functional polymer nanostructure materials [49,50]. As it has been already discussed by Rovigatti and co-workers [51], rather simplified models featuring hard spherical particles and short-range patch-patch attractions have been used to establish the majority of the theoretical breakthroughs in describing the complex nature of the assembly behavior of soft patchy particles.

The study of intra- and intermolecular assembling of soft Janus and patchy particles becomes extremely challenging as their sizes involve both the microscopic and mesoscopic scales (typically, their sizes range from 10 nm to 1000 nm, and even up to the micrometer scale) [52]. In the case of hard patchy particles, several generic models have been successfully developed including the extended Kern and Kern-inspired patchy models [42,53,54,55], the spot-like patchy models [43,44], and the rigid-body patchy models [56,57]. On the other hand, in the case of soft Janus and patchy particles, because of their more complicated architecture, mesoscopic coarse-grained methodologies are better suited [58,59,60,61,62,63,64,65,66]; however, nowadays, there is still not a generic mesoscopic coarse-grained model to enable capturing the aggregation behavior of various types of soft Janus and patchy particles.

The studies carried out by employing generic coarse-grained models do not probe directly the effect of different chemistry, and the interaction between the different components is usually controlled by a set of adjustable parameters. Advanced detailed simulation techniques (that could capture the atomistic level) are instead required to be able to address with high accuracy the microstructural and dynamical features of complex patchy particles as a function of the external environmental conditions such as temperature, pH, ionic strength (salt concentration) and host–matrix (solvent), and as a function of the fine details of the chemical constitution of patchy particles (e.g., chemistry of the arms, local packing, etc.). Both the accurate representation of monomeric characteristics and the intermolecular interactions are important to specify the structure of specific multi-component polymer materials. Recently, we presented a protocol for designing patchy-like morphologies by internal nanosegregation of mikto-arm stars [67]. More specifically, we showed that a single-molecule mikto-arm star with 16 equally long, immiscible poly(ethylene oxide) (PEO) and polystyrene (PS) arms forms a patchy-like structure in vacuum and in polybutadiene (PB) host (i.e., in the environment mimicking bad solvent conditions) [67]. The effect of the number of arms and their arm length in (PS/PEO) mikto-arm stars as well as the interaction with different host environments was also examined, resulting in a variety of unimolecular nanosegregated objects with internal morphologies ranging from Janus-like to “octopus-like” [67]. Concerning the dynamics of the inner segments in the mikto-arm stars resembling the patchy particles, we showed that, in the central region of the star, where the arms are in close contact due to geometrical constraints induced by their attachment, the segmental dynamics is analogical to that found in dynamically asymmetric miscible polymer blends or disordered copolymers [68]. In other words, the heterogeneous local environment caused by the packing of the segments inside of the 32-arm (PS/PEO) star was closely related to the presence of domains with mutually correlated dynamics.

Motivated by our recent studies of self-assembling behavior of mikto-arm stars in a selective oligomeric matrix [69,70], we turned our attention from a single-molecular patchy-like system described above to the multi-molecular assemblies of these building units. More specifically, we present here a simulation study of intra- and intermolecular nanosegregation of mikto-arm stars consisting of 16 PEO and 16 PS arms, (PS)${}_{16}$(PEO)${}_{16}$, in an environment in which they preserve their patchy-like morphology. Note that, besides the immiscible character of PEO and PS arms, these two star components are also dynamically and structurally contrasting polymers; this means that they differ significantly in their flexibility and in their glass transition temperature [71]. When combined in mikto-arm star architecture, their contrasting character was found to be highly beneficial in pursuit of devising high conductivity, high modulus solid polymer electrolytes [72]. Polybutadiene matrix was chosen as a commonly available medium in which the PEO arms form more than one segregated region per star [67]. The compatibility of PS/PB linear mixtures has been studied for lower temperatures and higher molecular weights than those presented here [73,74,75], reporting poor miscibility of PB and PS polymers. However, as the conditions used here regarding the molecular weight and the temperature are identical to those used in our single-star study reported in Ref. [67], where we did not observe any segregation of the PS arms in the PB environment, segregation of the PS arms in the PB matrix in the current study is not expected. Aware of the computational challenges associated with the extension of our study from the single-molecular to the multi-molecular systems, we systematically build up our computationally designed samples, varying the number of “building units” from 2 to 8. As we keep all parameters such as number of arms, their length, external conditions (i.e., temperature and the polymer matrix), and weight fraction of the stars constant, the only difference between the samples is the number of mikto-arm stars participating in the self-assembled structure. This protocol allows us to examine in detail the soft and directional interactions that arise between the patchy regions. To our best knowledge, this is the first attempt to examine the assembling behavior of soft, internally nanosegregated patchy particles preserving all their chemistry-related features by applying atomistic molecular dynamics simulations. Taking advantage of the given method, we aim to describe specific chemistry and local properties, such as packing in segregated domains, in order to build a solid base for the future usage of mikto-arm stars as potential building blocks in nanosegregated materials.

## 2. Models and Methods

**Figure 1:**
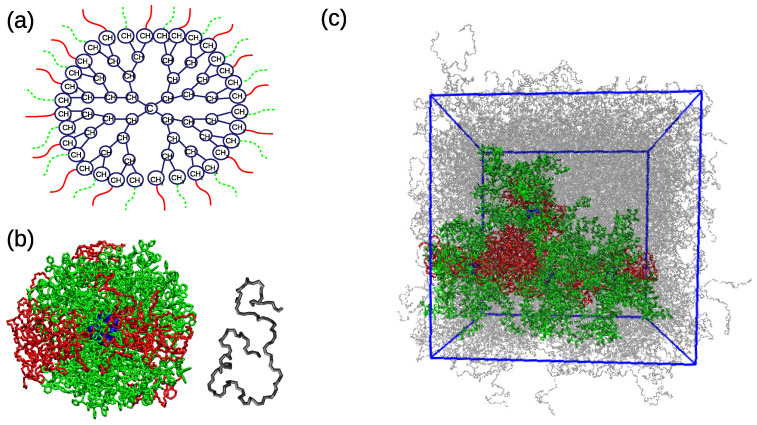
Illustration of the system’s components and the simulation system. (**a**) Drawing of the central dendritic core; (**b**) initial configurations of a mikto-arm star and a PB chain. The PEO arms are painted red, the PS arms green, the central core blue. (**c**) A representative snapshot of an equilibrated s4/PB system. The PB matrix is transparent for a better visualization.

We studied self-assembly processes in the samples of mikto-arm stars resembling patchy-like particles in an environment, where the patchy-like internal morphology is preserved. More specifically, the simulated systems consisted of $n_{s}$ mikto-arm stars embedded in the linear cis-1,4-polybutadiene (PB) matrix. The mikto-arm star was created by attaching 16 poly(ethylene oxide) (PEO) and 16 atactic polystyrene arms to a dendritic core structure in an alternating way (Figure 1a). Each arm is 40 monomeric units long, i.e., the composition of the studied stars can be summarized as (PS${}_{m}$)${}_{n}$(PEO${}_{m}$)${}_{n}$, with m=40 being the number of monomeric units per arm and n=16 being the number of arms of each polymer type. The PEO arms are terminated by a methyl group. The central core is built by 61 carbon (CH${}_{2}$, CH, C) units, and it has a dendritic architecture of four generations. The PEO and PS components differ in flexibility and due to very distinct features of their monomeric units also in the local packing when built in a star-like architecture. Estimation of these two characteristics in a highly heterogeneous environment, such as the one studied here, is a non-trivial task; however, recently, we showed that, in the homopolymer analogues of our mikto-arm stars, more flexible PEO arms packed closer to each other in the 32-arm PEO star than the PS arms in 32-arm PS stars in melt, due to the bulky character of the side groups (aromatic rings) and the related geometric constraints in the PS stars [76]. For more information about the local packing and dynamics of the PS and PEO arms in single-component, homopolymer stars in melt conditions, we refer readers instead to our recent studies [76,77]. Linear cis 1,4-polybutadiene chains containing 30 monomeric units are blended with the stars. The PEO arms in the (PS)${}_{16}$(PEO)${}_{16}$ mikto-arm stars nanosegregate into multiple domains in the PB environment at the given temperature, similarly to the case of a single mikto-arm star in vacuum (i.e., in the “bad solvent” condition for both star components) [67], owing to the generally immiscible character of PEO with PS and PB [78,79].

The weight fraction of stars in the matrix was held fixed at 15% for all systems studied here. We varied the number of stars in the system, $n_{s}$, to have full control of the number of building blocks in the self-assembled macromolecular object, in order to study the mutual orientation of the patchy regions. We chose notation s$n_{s}$/PB to stress the composition of each blend, namely, notation s6/PB means that there are six (PEO${}_{40}$)${}_{16}$(PS${}_{40}$)${}_{16}$ stars embedded in the linear PB matrix, whose weight fraction is 15%. A single-star system s1/PB with the star weight fraction of 15% was used as a reference system. In some sections, for the sake of broadening the information about the self-assembly behavior and local packing of the stars, we compare the mikto-arm stars studied here, i.e., those in the PB matrix, with the identical stars in a selective polymer matrix, namely oligomeric PEO [70]. The weight fraction of those stars is 33%, and they form a percolated network in the oligomeric PEO matrix.

The preparation (generation and equilibration) of the *single-molecule mikto-arm stars* can be found elsewhere [67,68,69,70]. We recall briefly that fully-stretched arms are attached to the core, the internal structure is relaxed through various short simulations which also include energy minimization procedure, and, once there are no overlaps and/or stretched bonds, the star is equilibrated in the given medium.

The preparation of *the multi-star/linear blends* was done as follows:

1. Fully collapsed stars prepared in vacuum (see Figure 1b and Ref. [67] for more details) were randomly placed in the box; the box size was chosen to be just big enough for the given number $n_{s}$ of stars to fit inside without overlapping.
2. The space around the stars was filled with randomly oriented PB chains; due to the random orientation and random placement of the chains and the stars, there were voids between the molecules, and the density was much lower than the one of a fully equilibrated system. The fraction of the chains added in this step varied depending on the system; it was around 1/2 of the overall mass of the matrix for the s2/PB system and 1/4 for the s8/PB system.
3. A very short NPT simulation of around 50 ps was performed to slightly orient the PB chains and make space for the insertion of more chains of the matrix. The velocity-rescale algorithm was used to maintain the temperature at 600 K and the Berendsen barostat to keep the pressure at 1 atm. The time step was 0.1 fs. Due to the chosen high temperature and incompressibility of the PB matrix, the small voids got progressively filled by the quickly rearranging PB matrix and the barostat did not lead to any abrupt deformation of the system. In multiple iterations, we inserted the given number of chains, i.e., the number which corresponds to the star weight fraction of 15%. Notice that at this stage the system still contained heterogeneities in density (i.e., small voids between randomly placed items).
4. To reduce heterogeneities in density in the blend of stars and linear chains, we run a longer run (few nanoseconds) with the same settings as in step 3 until the box size fluctuated around an average value. Note that, in principle, it is possible to insert randomly the required number of chains at once in the step 1 and 2 above (i.e., to skip the step 3), if the box size is enlarged to fit all molecules; however, such initial configuration leads to numerical instabilities, as the differences in density in different parts of the box are huge, which causes a malfunction of the barostat.
5. Once the box size fluctuated around an average value, we changed the time step to 1 fs and continue the equilibration for another 20 ns.
6. We performed a run of 40 ns using Langevin dynamics at 500 K. The random component of the Langevin thermostat helps overcome possible barriers related to the rearrangement of the system components.
7. A cooling run of 20 ns was performed to reduce the temperature from 500K to 400K.
8. We fixed the bonds with the LINCS algorithm [80] and performed the pre-production run. The temperature T=400 K was maintained by the Nose–Hoover thermostat and the pressure of 1 atm by the Parrinello–Rahman barostat. The length of the pre-production run varied with $n_{s}$, from 155 to 185 ns.

During the equilibration, the system components of different polymer types (i.e., PEO, PS, PB) were coupled to separate thermostats to avoid creation of stationary temperature gradients [69,81]. The Coulomb interactions in steps 1–7 were described by the cut-off scheme with a cut-off distance of 1 nm, and the pre-production run in step 8 was performed with the particle-mesh Ewald (PME) method for the electrostatics. The production run was 50 ns long, and it employed the same settings as in step 8 of the equilibration. A snapshot of a fully-equilibrated s4/PB system is shown in Figure 1.

Except for this main preparation protocol, an alternative method for preparing multi-star systems with six and eight stars was also considered in this work, starting from a different (independent) initial molecular configuration, to assess the sampling performance attained from two different generation/equilibration approaches. In the second approach, instead of starting by placing the stars randomly into the box, we started from a collapsed single-star system initially prepared in vacuum, and we replicated it along the *x*, *y*, and *z* by adding one extra star at a time. Following this procedure, the stars were distributed in space in a periodic way; this means as if the image of the single star was placed in the points of a crystal lattice. Then, a short NPT simulation was performed in vacuum to reduce the distances between the stars. The time step used was 0.1 fs and the temperature was 600 K. Once the stars formed an assembly and distances between the stars fluctuated around their average values, the PB chains were inserted in the box in a step-wise manner similarly to step 2 above. Then, the procedure continued with steps 4–8 above. Taking into account the external conditions during the preparation of the system, this second method would be similar to an experimental protocol which would start with a preparation of the sample in a bad solvent (here mimicked by vacuum) and then blend the obtained assembly in the PB matrix. Therefore, we will refer to it as the “assembly-first” approach. On the other side, in the first stages of the main preparation protocol presented above (steps 2–4), the particles are well dispersed; therefore, we refer to this approach as a “dispersed-first” approach. This approach would mimic an experimental situation where the sample would be first prepared in dilute conditions and then the remaining solvent would be removed in successive steps in order to achieve a well dispersed state. In what follows, we present the properties of the computationally prepared samples following the “dispersed-first” approach and discuss the differences in both preparation methods in the Discussion section. In order to distinguish the systems prepared by the “assembly-first” approach, we named them cs6/PB and cs8/PB.

All presented simulations were performed with the Gromacs simulation package [82] and the snapshots created by the VMD graphics software [83]. The polymers were modeled by the united-atom model of the TraPPE force field [84,85,86]. The united-atom approach does not treat the hydrogens explicitly, but combines them with the carbon atom in one united-atom unit, i.e., CH${}_{2}$. Only the PEO united atoms carry partial charges and thus only PEO arms contribute to the electrostatic energy [86].

## 3. Results

### 3.1. Properties of the Building Blocks: Intramolecular Analysis

As we reported in our former work [67], the mikto-arm stars under study internally nanosegregate into patchy-like particles in the PB matrix at the given temperature (note that the single-star system reported in Ref. [67] has a slightly lower weight fraction of the mikto-arm star in the matrix). The PEO arms form segregated domains while the PS stars do not indicate any preferable orientation in space; thus, they do not aggregate at the given conditions [67]. In order to study systematically the simulated systems, we first focus on the properties of the individual stars, thus the intramolecular properties of our building blocks, and verify that the building blocks maintain their properties reported for the single-star simulations.

One way to characterize the size and consequently the shape of the studied patchy-like particles is to measure the radius of the gyration tensor. The elements of the radius of gyration tensor $S_{\alpha \beta }$ are defined as:

(1) $$S_{\alpha \beta }=\frac{1}{N}\sum_{i=1}^{N}\left(r_{\alpha }^{i}-r_{\alpha }^{cm}\right)\left(r_{\beta }^{i}-r_{\beta }^{cm}\right)$$

where α and β represent the *x*, *y*, or *z* components of the Cartesian coordinates, *N* is the total number of atoms in the entity under study (in our case, either the whole star molecule or the star arms), $r_{i}$ is the position vector of atom *i* and $r_{cm}$ is the position vector of the center of mass of the entity. The radius of gyration of the star Rg or of the arm $Rg_{a}$ can be then calculated as the trace of the given tensor, $Rg^{2}=\lambda_{1}+\lambda_{2}+\lambda_{3}$, where $\lambda_{1,2,3}$ are the eigenvalues of the tensor and $\lambda_{1}\leq \lambda_{2}\leq \lambda_{3}$. The average values of the radius of gyration $Rg=\sqrt{<Rg^{2}>}$ and the arm radius of gyration $Rg_{a}=\sqrt{<Rg_{a}^{2}>}$ obtained from the simulations are summarized in Table 1. The size of the individual stars in the multiple-star systems is comparable to the size of the reference single-star system, only a negligible increase in Rg was found in the former. The size of the PEO and PS arms is identical in all systems at the given accuracy. In other words, no significant change of the size of the stars and the corresponding star arms was observed when the stars self-assemble.

To characterize the shape of the mikto-arm stars and their arms, we calculate the asphericity parameter through:

(2) $$a=\frac{{\left(\lambda_{2}-\lambda_{1}\right)}^{2}+{\left(\lambda_{3}-\lambda_{1}\right)}^{2}+{\left(\lambda_{3}-\lambda_{2}\right)}^{2}}{2{\left(\lambda_{1}+\lambda_{2}+\lambda_{3}\right)}^{2}}$$

**Figure 2:**
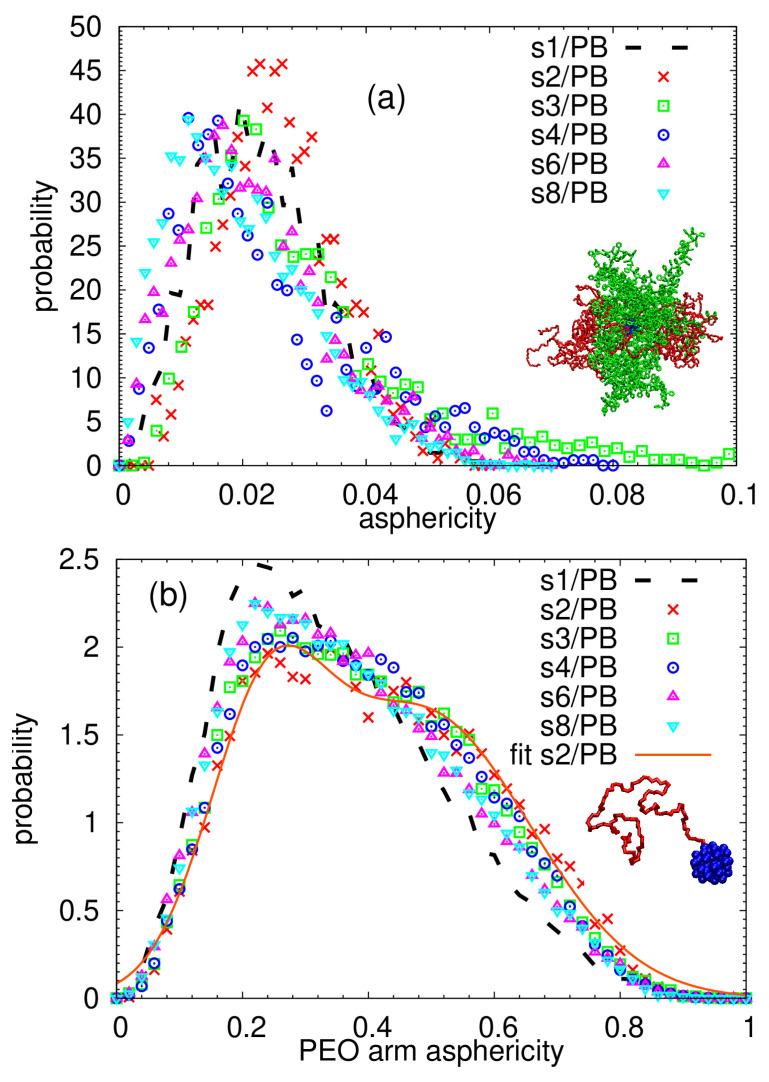
The shape parameters of the individual stars in the assembly: the distributions of (**a**) the star asphericity and (**b**) the PEO arm asphericity together with a randomly selected snapshots of (**a**) a star and (**b**) a PEO arm connected to the core chosen from the s3/PB system. The solid line in (**b**) represents the best fit of the s2/PB data to a sum of two Gaussian functions.

Note that a perfectly spherical object has asphericity equal to a=0. The probability distribution functions for the stars and the PEO arms are plotted in Figure 2. As also seen from the snapshot of a randomly selected star of s3/PB system (Figure 2a), the mikto-arm stars are fairly spherical and only minimal shape fluctuations are detected among the systems. A similar conclusion can be made when comparing the probability distribution functions of prolateness, another shape parameter, which is shown and described in Figure S1 of the Supplementary Materials. This similarity in the particle shape in the single-star and multiple-star systems is in contrast with the behavior of analogous 32-arm mikto-arm stars in a selective, oligomeric PEO matrix, where a more prolate shape was reported for the stars forming a self-assembled object than for an unimolecular system [70]. It is also important to mention here that, in the selective PEO matrix, the final structure is a result of an interplay between two driving forces which cause a close packing of the segregated stars: (i) the tendency to maximize the contact between the PEO arms and the PEO matrix and (ii) the tendency to avoid the unfavorable PS:PEO interaction [70]. The patchy particles studied here arrange into anisotropic macrostructures to maximize the intermolecular contacts of the PEO arms, and this aggregation does not cause the deformation of their overall shapes, only a space reorientation, as shown later on.

Concerning the asphericity of the PEO arms, subtle differences can be observed in Figure 2b. It has been shown that, in the case of the homopolymer stars in the θ conditions or in the melt, the distribution of the arm asphericity is bimodal, with each peak representing a characteristic arm conformation [76,87]. More specifically, it can be approximated by two Gaussian functions, the position of the first maximum corresponds to the collapsed conformation and the second one with the higher values of asphericity to the coil [87]. As this feature seemed to reflect subtle fluctuations of the local conformation in the coarse-grained [87] as well as in the atomistic model of the homopolymer stars [76], we fitted our distributions to a sum of two Gaussian functions to examine whether the PEO arms in our complex multicomponent systems behave as if they were in a much more homogeneous, ideal θ state. One example of such a fit is shown in Figure 2b for the s2/PB system. The parameters of the fit (i.e., the positions of the peaks, weights of each Gaussian function and the respective dispersions) are almost identical to those found in Ref. [76] for the melt of 32-arm PEO stars at 450 K. The sum of two Gaussian functions approximates our data very well also in the case of s3/PB stars; it fails for the single-star system, where the distribution is clearly shifted towards lower values of arm asphericity, indicating predominantly collapsed conformation of arms. The collapsed state detected from the PEO arm asphericities of s1/PB is in agreement with the results reported in Ref. [67], where a morphological and conformational resemblance of a single star in the PB matrix and in vacuum (i.e., a bad non-solvent environment) was found. As the number of stars in the system increases, namely becomes higher than 3, the distributions of the arm asphericities are deviating from the bimodal character and approach the distribution measured for single-star s1/PB. In other words, when two and three patchy particles are interconnected, their PEO arms fluctuate between two configurational states reflected by the bimodal distributions of their asphericities, an indication of a more homogeneous local environment (i.e., an environment mostly composed of surrounding PEO arms), while the PEO arms of the individual stars in systems consisting of more than three building blocks seem to preferentially adopt the collapsed configuration.

On the other side, the probability distribution functions of asphericities obtained for the PS arms merge for all studied systems and their main and only maximum is shifted to higher values of asphericities, indicating a more prolate shape than the one found for the PEO arms (see Figure S2 in the Supplementary Materials).

**Figure 3:**
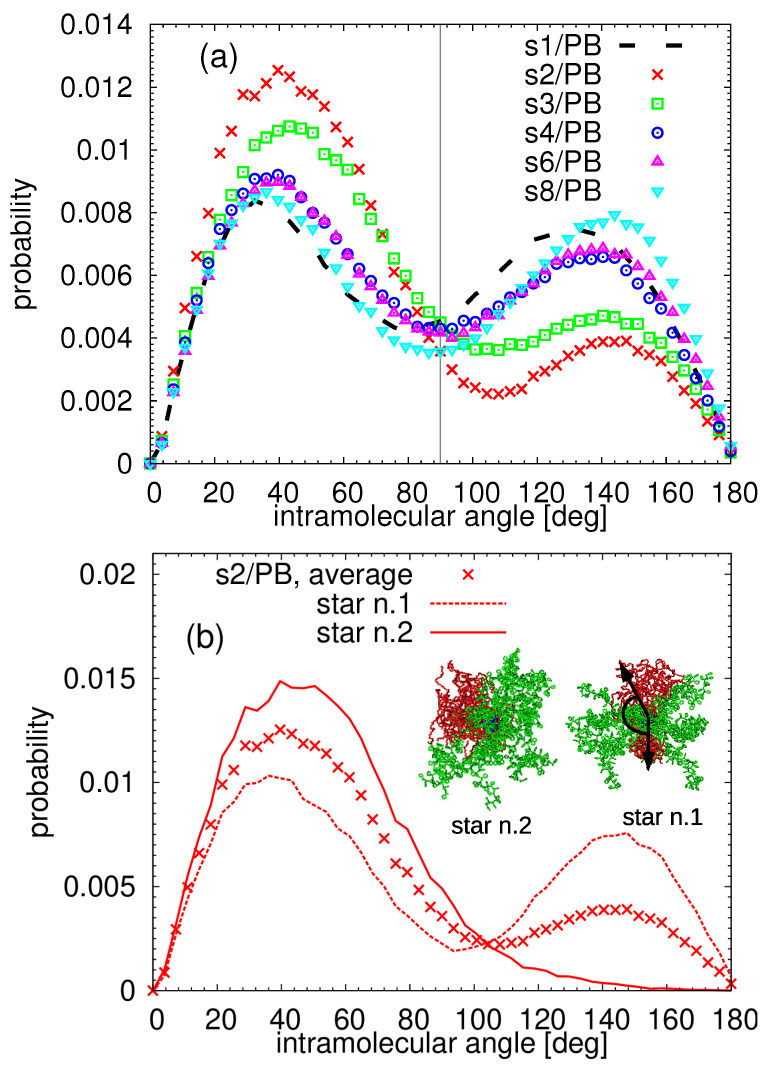
The intramolecular space arrangement of the PEO arms: the distributions of the angles among all inner center-to-end vectors of the PEO arms. Two examples of the center-to-end vectors are illustrated by the black arrows, the angle between them by a black solid line. In (**a**), the distributions are averaged over all the stars in the system, in (**b**), the solid and dashed lines represent the data calculated only for a specific selected star of s2/PB system. A representative snapshot of a selected star is also included. The vertical grey solid line in (**a**) marks the 90${}^{\circ }$ criterion for the algorithm detecting the patchy regions.

To identify the internal morphology of our building blocks, we measured the mutual orientation of the arms inside the particle; a protocol introduced in Ref. [67]. The orientation of each arm was defined by assigning a center-to-end vector to it, it means the vector connecting the central part of the star (i.e., the central carbon of the dendritic structure, see Figure 1) and the terminal end atom on each arm. A schematic illustration of this vector can be found in the inset of Figure 3b. Then, we measured all angles among the vectors of the arms of the same chemistry belonging to the same molecule, i.e., among 16 PEO and 16 PS arms in each star. The probability distribution functions averaged over all internal angles related to the PEO arms in the systems are presented in Figure 3. As explained in detail in Ref. [67], when arms internally nanosegregate, they form domains in which the arms are aligned close to each other, which leads to a peak in the distribution function placed at low values of the angles. Additional peaks corresponding to obtuse angles indicate a presence of more than one segregated domain, and thus it is a characteristic feature of the patchy-like morphology. As readily seen in Figure 3a, the building blocks in each system form patchy-like structures internally. The patches in the single-star s1/PB system seem to be positioned at a slightly lower angle than in the multiple star systems, which can be related to the fact that the position of the internally segregated regions in the multiple-star systems is affected by the intermolecular interactions. Interestingly, in the case of the s2/PB and s3/PB systems, the second characteristic peak is smaller than in the remaining systems that can either mean less populated patchy regions or, in a limiting case, a presence of only one segregated domain in some particles.

To further examine the above issue, we calculated the probability distribution function for each star in the system separately and plotted the results in Figure 3b for the s2/PB system. Indeed, to maximize the PEO:PEO contacts, one star in the s2/PB system aligned its PEO arms in such a way that the molecule resembles an octopus, with the PEO arms being the “head” and the PS arms stemming from it (see the snapshot in the inset of Figure 3b). This arrangement of the arms leads to a single peak in the distribution function and thus, when averaged together with the second star, which maintains the patchy like morphology during the duration of the simulation, the final probability distribution functions exhibit two unequally populated peaks as in Figure 3a. In the s3/PB system, we detected one case of such an “octopus-like” morphology. All stars in the systems with more than three stars, namely s4/PB, s6/PB, and s8/PB, exhibit a bimodal distribution of the angles among the center-to-end vectors of the PEO arms, retaining their patchy-like morphology even when incorporated in a macromolecular assembly.

No preferential alignment of the PS arms in any of the studied systems was found (data not shown). The observations related to Figure 3 were confirmed by measuring the distances among the centers of mass of the arms of the given chemistry: the PEO arms packed closely in the segregated regions, giving rise to a main maximum in the distribution function at very short distances while the arrangement of the PS arms did not lead to well distinguished maxima in the radial distribution functions. The radial distribution functions for the PEO and the PS arms are plotted as Figure S3a,b of the Supplementary Materials, respectively.

**Figure 4:**
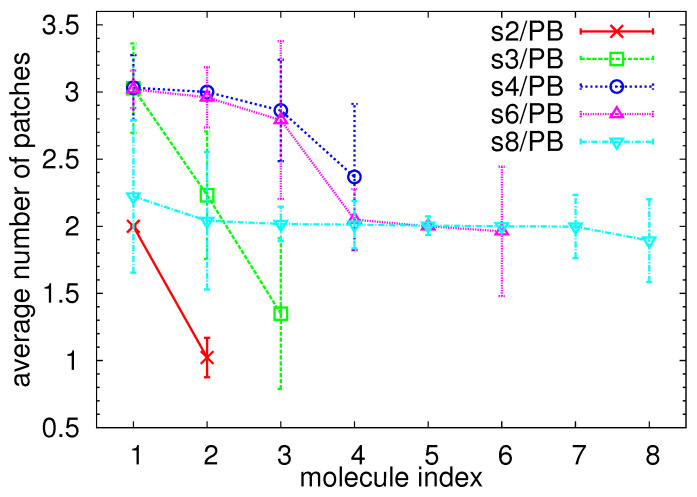
Average number of patches per star plotted as an index of the star in the system. Stars were sorted and labeled in descending order of the number of patches. The lines are guide for the eye.

**Figure 5:**
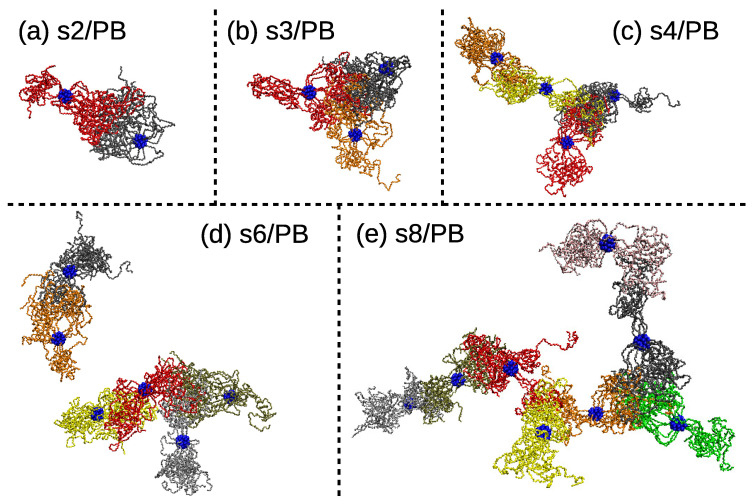
Randomly selected snapshots of the PEO domains aggregated in the macrostructure in (**a**) s2/PB, (**b**) s3/PB, (**c**) s4/PB, (**d**) s6/PB, and the (**e**) s8/PB system. Domains belonging to different stars are painted with different colors, and the central dendritic structure of each star is depicted with blue spheres.

The distribution functions showed in Figure 3 serve as a tool for distinguishing the patchy-like morphology from the “octopus-like” one; however, they lack the information about the number of patchy regions per star. Note that the identification of the different segregated domains is a very challenging task also in experiments, as recently reported for heterografted hairy nanoparticles [88], which in the limit of a small core size to graft length ratio share multiple features with mikto-arm stars. In order to identify these regions in each star, we came up with an algorithm which allocates arms into different regions according to their mutual orientation. Note that an allocation according to their mutual position (e.g., building on the results shown in Figure S3 of the Supplementary Materials) is impractical, as, due to the alternate attachment of the arms, the arms are mixed in the vicinity of the dendritic core. First, we define a vector from the central atom of the dendritic core to the center of mass of the arm $v_{i}$, for each arm i=1,2,..,16. Consequently, we calculate the angle $\theta =\arccos\left(\frac{v_{i}\cdot v_{i+1}}{∥v_{i}∥∥v_{i+1}∥}\right)$ between the corresponding vectors of the consecutive arms and join them into the same patch if the condition $\theta <90^{\circ }$ is satisfied. The angle 90${}^{\circ }$ was selected as a result of the analysis shown in Figure 3; this angle seems to be a characteristic angle of the arms packing in one common domain. We also varied this angle in a range 45${}^{\circ }$–115${}^{\circ }$ to test the versatility of our algorithm; the results of the test are summarized in the Supplementary Materials. The algorithm detects the number of patchy regions for each star at a given time. Due to thermal fluctuations of relatively flexible PEO arms and the fact that the patchy domains are not fixed in space, as is the case of patchy particles produced by the surface modification, the number of patchy regions per star may slightly change during the simulation as the arms rearrange and adapt to orient towards the nearest neighbor particle. We calculated the average number of patches for each star $n_{p}$ in the simulation time window, sorted the stars according to this number, starting with the labeling with the star with the highest number of segregated domains and plotted the results for each star in descending order in Figure 4. The change in the internal morphology is reflected in the error bars, i.e., in the molecules with small error bars, the arms do not rearrange significantly. We observed that the rearrangement of the PEO arms is facilitated in the poorly populated “loose” patches, i.e., patches that do not participate in the “link” between the stars and contain only a few arms, as those arms tend to either orient to form a “link” or align with the remaining patches of the molecule to avoid the unfavorable interaction with the PB matrix. As already commented in Figure 3b, the systems s2/PB and s3/PB contain one star with arms predominantly arranged in an “octopus-like” pattern, having the $n_{p}<2$. The particles in the remaining systems encompass on average 2 or 3 patchy domains, which is in agreement with the observations in Figure 3 and with the visual inspection of the snapshots in Figure 5 (recall that a patchy particle with more than two patchy regions regularly distributed in space can also exhibit bimodal distribution of the angles among the center-to-end vectors of the PEO arms).

### 3.2. Properties of the Macromolecular Assembly: Intermolecular Analysis

The snapshot of the s4/PB system shown in Figure 1 can serve as the first evidence that the stars are connected into anisotropic structures. The main driving force of the intermolecular segregation is the tendency of the PEO arms to avoid the unfavorable interaction with the PB matrix. In the multi-star systems, the stars orient in the space in such a way that the contacts between the PEO arms of the neighboring stars are enabled, reducing the number of monomers of the PB chains in their surroundings (see the local number density of the monomers of the PB matrix around the PEO and PS monomers in Figure S4a,b of the Supplementary Materials, respectively).

In order to get a better picture of the way the patchy regions link together, we selected only the PEO arms and the central dendritic core and visualize them in Figure 5. The main feature obvious at first glance is that the patchy regions are interconnected. It means that the patches are not aggregated in isolated clusters, as is the case for the patchy particles designed by internal nanosegregation of telechelic stars [58,59,89] or grafted nanoparticles [2,90,91], but one can follow the arm contour and continuously move from one patch to another. Note that, due to this feature, we could not apply standard cluster analysis algorithms to estimate the number of nanosegregated regions in the nanoparticles, and we opted for the algorithm presented above. In the s2/PB system, the “octopus-like” particle connects with one of the patches in the neighbor star and forms a straight, rod-like object. As the number of stars in the system increases, it is possible to identify more complex patterns in the interconnected structure.

**Figure 6:**
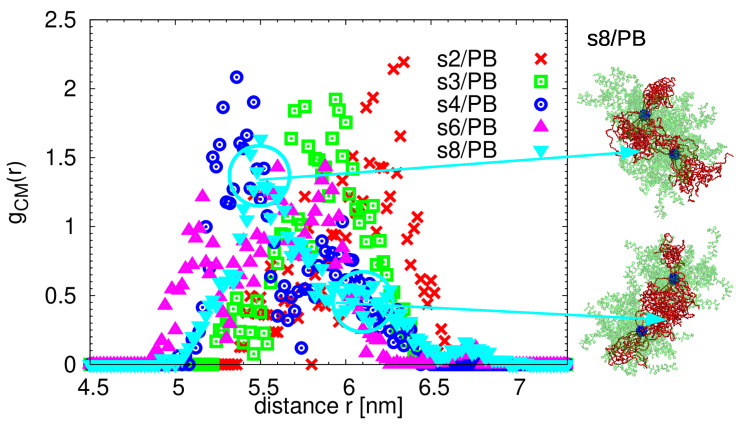
The radial distribution function of the mutual distances of the centers-of-mass of the nearest neighbor stars in the self-assembled object. Representative snapshots for selected distances highlighted by circles are shown on the right for the s8/PB system.

As the first step is to analyze these patterns, we look at the interpenetration of the nearest neighbor stars. As can be seen from the plot of the normalized radial distribution functions of the centres-of-mass distances of the nearest neighbor stars in the self-assembled object in Figure 6, more than one characteristic distance can be detected. The degree of interpenetration is mostly determined by the packing of the PS arms, as their bulky side groups (aromatic rings) impede the access of the arms of the surrounding stars. We verified this statement by checking the same property in the homopolymer PS and PEO stars in melt. The closest distance between two neighboring stars in 32-arm PS stars in melt is identical to the one found here (around 4.8 nm), while, in the case of analogical PEO stars, this distance is much smaller (data not shown here). The interpenetration of the stars is only shallow, as many of the simulated stars are separated by distances comparable to the sum of their Rg (see Table 1 and notice the population of the stars at distances around 5.8–6.3 nm in Figure 6). Concerning the orientation of the PEO patches, the horizontal alignment of the patches was mostly found in the stars that are positioned at distances larger than 6 nm. On the other side, stars that manage to interpenetrate more have their patchy domains connected in the T or L shape (see snapshots in Figure 6). We would like to stress that, despite the fact that the patches can freely reorient and rearrange inside of the molecule, building blocks in the assembly do not change their “linking” partners; they only slightly adjust their mutual positions and this motion results in broad and noisy distributions in Figure 6. Having in mind that the position of the center of mass is vastly governed by the space arrangement of the PS arms (they have higher molecular mass), in what follows, we will use the core–core distance to identify the nearest neighbor stars.

**Figure 7:**
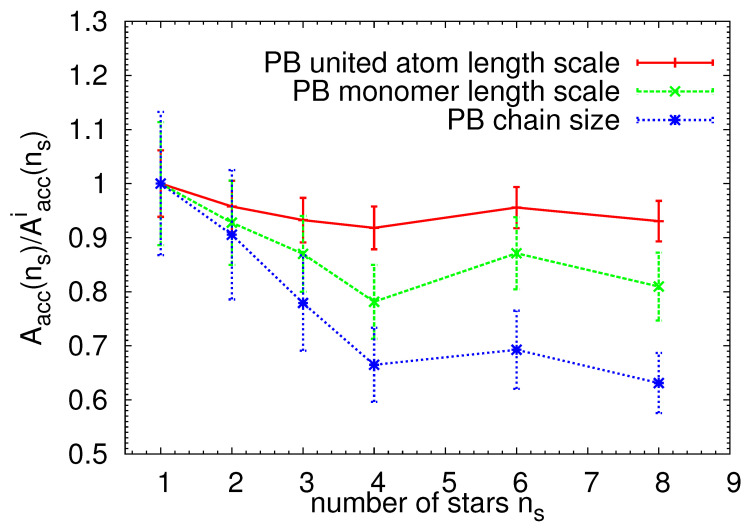
Accessible surface area $A_{acc}\left(n_{s}\right)$ normalized with a value for the ideally dispersed system $A_{acc}^{i}\left(n_{s}\right)$ as a function of number of stars in the system, $n_{s}$. The accessible surface area for the ideally dispersed system was calculated as $A_{acc}^{i}\left(n_{s}\right)=n_{s}A_{acc}\left(1\right)$, where $A_{acc}\left(1\right)$ is the value obtained for s1/PB. Different symbols represent different radii of the “solvent” probe, i.e., in our case, different characteristic length scales of the PB matrix. The lines are guide for the eye.

The degree of the penetration of the stars in the system can also be seen from the plot of the normalized accessible surface area $A_{acc}\left(n_{s}\right)$ presented in Figure 7. We obtained the accessible surface area by applying the algorithm of Eisenhaber et al. [92] implemented in the Gromacs simulation package [82]. Briefly, the double cubic lattice method is used to find the part of the surface area of the stars exposed to the PB molecules. An important parameter of the calculation is the radius of the “solvent” probe, the smaller the radius, the higher the probability of accessing the surface of the molecule. As in our case the building blocks are dispersed in the polymer matrix, we use three characteristic length scales as the estimation of the probe radius: one of the order of the sizes of the PB united atoms, one comparable to the monomer size, and one comparable to the chain size. The results were normalized by the accessible surface area of the ideally dispersed system $A_{acc}^{i}\left(n_{s}\right)$, which is defined as $A_{acc}^{i}\left(n_{s}\right)=n_{s}A_{acc}\left(1\right)$, where $A_{acc}\left(1\right)$ is the accessible surface area of the s1/PB system, and $n_{s}$ is the number of stars in the system. Notice that, by choosing this representation, the plot can be directly related to the fraction of the accessible surface area of the star-like particles which was “lost” due to the linking and penetration of the building blocks, as no penetration would lead to a trivial $A_{acc}\left(n_{s}\right)/A_{acc}^{i}\left(n_{s}\right)=1$ dependence. As seen in Figure 7, if the probe radius is of the order of the size of the united atom, the dependency of the normalized accessible surface area on the number of stars in the system deviates only slightly from the ideally dispersed system. Having in mind that the particles maintain their spherical shape even after the self-assembly process (Figure 2) and that only a minimal change in the local environment of the PS monomers was found when comparing single- and multiple-star systems (see the local number density in Figure S4b in the Supplementary Materials), the results in Figure 7 confirm the observation made in Figure 6, indicating that the particles do not fully interpenetrate; instead, they form “links” only by suitable orienting of the patchy regions. Note that the low penetration degree is related to the unentangled character of the star arms in combination with the high star functionality. We therefore expect similar behavior to the one presented here in systems with analogous softness without the topological (entanglement) effects. Mixed grafted nanoparticles [88,93] or recently synthesized mikto-brush-arm star polymers [94] may serve as an excellent example of systems with analogical internal packing of the system components. As indicated in the recent experimental [95] and simulation studies [76,77], such tuning of the softness can be achieved by adjusting three factors: arm length, number of arms (or grafting density), and polymer chemistry. In addition, the results shown in Figure 7 confirm that the stars in the studied systems do not form one, closely packed isotropic object (e.g., a ball-shape assembly), as such an object would give rise to a significant drop in the accessible surface area. Notice that, recently, a more complex geometrical analysis has been proposed to describe the aggregate morphologies formed by anisotropic self-assembly of polymer grafted particles [90,96]. However, the analysis protocol in these works was developed for particles with patchy regions located only on the surface of the particle, and thus is not applicable in our case.

**Figure 8:**
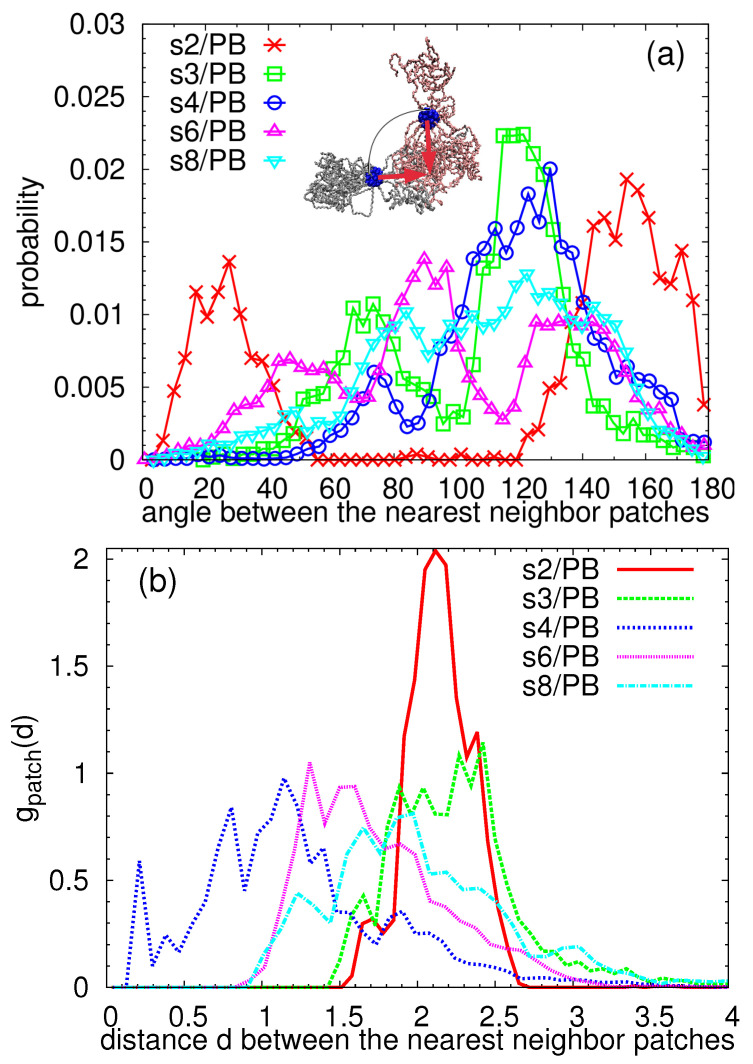
(**a**) The probability distribution function of the intermolecular angles (scheme in the inset) between the nearest neighbor patches; (**b**) radial distribution function of the intermolecular distances between the centers of mass of the nearest neighbor patches.

To look even closer at the mutual orientation of the PEO arms which form the link between the particles, we detected the patchy regions by the algorithm already described above and in the Supplementary Materials, we calculated the center of mass of each patchy domain and defined a vector connecting the central carbon in the dendritic core and the center of mass of each patchy region (red arrows in Figure 8a). Then, we selected a patch, found the closest patch placed in the surrounding stars, and calculated the mutual orientation of their vectors. The probability distribution functions of the angles between these vectors are plotted in Figure 8a for each system. Note that two patches aligned in one line opposite to each other would form a straight angle (180${}^{\circ }$), while the patches which do not contribute to the link (loose terminal patches) would form with the highest probability acute angles with the patches in the nearest neighbor star. Therefore, we attribute the obtuse angles to the linking patches. System s2/PB can serve as a simple example of such a case: the maximum at around 160 degrees reflects an almost straight alignment of the linking patches while a small peak at around 30 degrees belongs to the terminal, unlinked patchy region (few red unlinked arms in Figure 5a). The arrangement of the patches in s3/PB system can be also easily understood if the distribution function is broken down into the contributions from the individual stars: the main angle of 120 degrees represents a Y-shaped interconnection of the three patches of three stars, the small peak positioned at 70 degrees is present in the stars which contain two patches, and one of them is unlinked. As the number of particles in the system increases, the peaks in the distribution function in Figure 8a get wider and the function even exhibits multiple maxima, which is a consequence of a presence of multiple patches and their more complex arrangement. The peak positioned at 120 degrees in s4/PB and s8/PB system indicates, similarly to the s3/PB system, a presence of a Y linking pattern, while a 90 degrees maximum in the s6/PB distribution reflects a T-shaped pattern.

The radial distribution function of the intermolecular distances between the nearest neighbor patches is plotted in Figure 8b. Interestingly, patches are packed close to each other in the systems where we detected the highest numbers of patches per molecule, namely in s4/PB and s6/PB (see Figure 4). We speculate that this correlation between the number of patches per star and their intemolecular packing is due to less arms participating in the patches. In other words, if the segregated region is formed only by few (2–3) arms, its ability to adapt and approach a similarly adaptable region is higher, thus resulting in smaller mutual distances, than if the same adaptation is expected from an “octopus-like” particle connected to a highly populated patchy region, as is the case of 2s/PB system arrangement.

**Figure 9:**
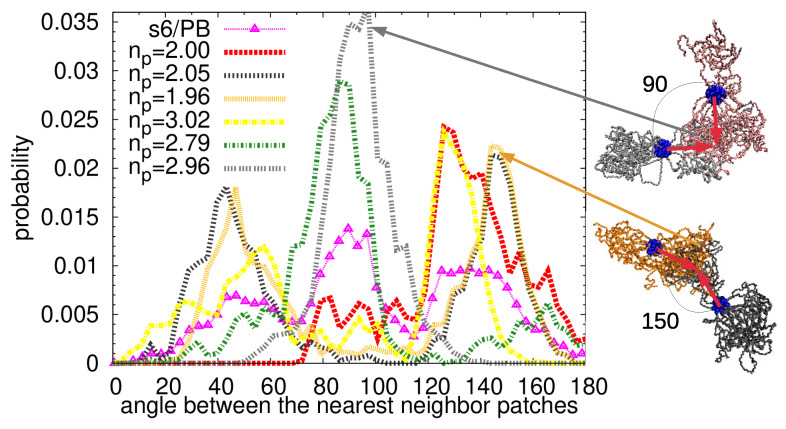
The probability distribution function of the intermolecular angles between the nearest neighbor patches for s6/PB system (magenta symbols). The distribution functions of the individual stars in the s6/PB system are plotted with lines, and the average number of patches per star corresponding to each star can be found in the legend. Characteristic snapshots of mutual configurations of two stars with angles between the patches of 90 and 150 degrees are shown on the right.

To investigate further the relation between the number of patches per particles and their mutual arrangements, we plot the probability distribution function of the intermolecular angles between the nearest neighbor patches averaged over all the particles in s6/PB system together with the individual functions for each particle in Figure 9. We also added in the legend the average number of patches $n_{p}$ in the corresponding star. Two stars with two patches per star aligned almost in a straight line can be spotted (the orange and dark grey lines in Figure 9). These two stars form a bimolecular object clearly seen from the snapshots in Figure 5d (the orange and dark grey molecule). In addition, two stars whose connected patches resemble letter T can be also distinguished (olive and light grey lines in Figure 9 and molecules of the same colors in Figure 5d), both having more than two patchy regions per particle. One distribution function does not contain angles smaller than 70 degrees (red line in Figure 9), which means that there are no loose terminal patches present in this molecule, and both patches contribute to the interconnected assembly, which is in accordance with the snapshot of this particle (red molecule in Figure 5d). The adjacent particle, painted with yellow in Figure 5d, and a yellow line in Figure 9 shares the main maximum with the star with $n_{p}=2.0$, as expected due to their connection; however, it also contains a small peak at 60 degrees and non-zero probability of the intermediate angles, which could be attributed to the rearrangement of the arms from the terminal to the linked patchy region.

## 4. Discussion

Owing to the soft character of the patches and the fact that they are internally interconnected, a huge variety of possible linking and orientation of the patches has been found. Starting from a random, well-disperse configuration, the linking process was fully directed by the diffusive and rotational motion of the particles (“dispersed-first” protocol in Section 2). To mimic different experimental procedures for a preparation of the samples, we also studied a case when the particles were first linked and then they diffused and rearranged into a final, stable anisotropic structure (“assembly-first” protocol in Section 2).

**Figure 10:**
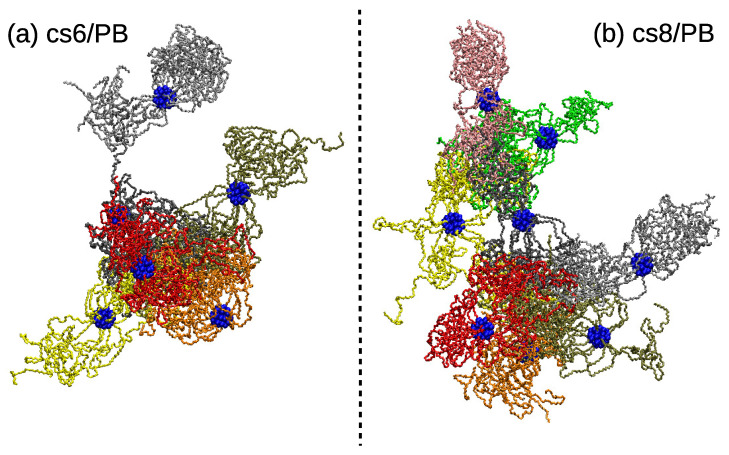
Randomly selected snapshots of the PEO domains aggregated in the assembly in (**a**) cs6/PB, (**b**) cs8/PB system prepared by the “assembly-first” protocol. Domains belonging to different stars are painted with different colors, the central dendritic structure of each star is depicted with blue spheres.

**Figure 11:**
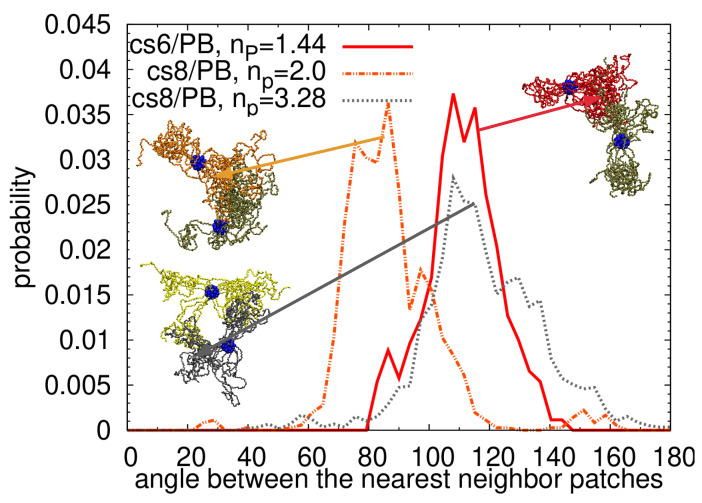
The probability distribution function of the intermolecular angles between the nearest neighbor patches for the star with the lowest number of patches in cs6/PB system (red line) and for the star with the lowest and the highest number of patches in cs8/PB system (orange and dark grey lines, respectively). The average number of patches per star corresponding to each star can be found in the legend. The arrows with the identical colors as the curves point to the characteristic snapshot of the link pattern.

The snapshots of the interconnected PEO arms in the assembly prepared by the “assembly-first” protocol are shown in Figure 10. At first glance, it is evident that the assembled structures are more compact than the assemblies prepared by “dispersed-first” protocol. The observation is in agreement with a lower local number density of PB matrix around the PEO and PS monomers shown in Figure S4 of the Supplementary Materials, confirming that the particles prepared by the “assembly-first” approach avoid more efficiently the contact with the matrix. Consequently, the accessible surface area was smaller for the cs6/PB and cs8/PB systems for all studied probe radii. The particles, however, retain their spherical shape and the behavior of the PS arms was identical to the systems prepared by “dispersed-first” approach, thus no internal deformation of the particles was spotted. The cs6/PB system contains two particles with a low number of patchy domains (Figure S7b in the Supplementary Materials) and therefore exhibits some similarities with the s3/PB system, where also 1/3 of the building blocks had less than 2 patches per star (see Figures S7a and S8b in the Supplementary Materials). Concerning the linking arrangement in these systems, despite the fact that the average distribution functions of the intermolecular angles between the patchy regions of cs6/PB and cs8/PB stars follow closely the data measured for the s8/PB system (Figure S8a in the Supplementary Materials), the different packing of the patchy regions (Figure S8b in the Supplementary Materials) suggests that there are diverse linking patterns present in the assembly. Indeed, we identified the patterns for the individual stars and found out that cs6/PB and cs8/PB systems preferably form a Y-linking pattern. The percentage of the patches aligned in line is lower than the one detected for the systems with six and eight stars prepared by a “dispersed-first” protocol. Moreover, we also found less peaks corresponding to loose terminal patches, which is in accordance with the above observations and results pointing out a more compact assembled structure in the samples prepared by the “assembly-first” approach. A few examples of the distribution functions for the individual stars can be found in Figure 11.

In summary, independently of the preparation method, the mikto-arm stars resemble spherical patchy-like particles, which, due to the unfavorable interactions of the patchy regions with the surrounding matrix, tend to link together and form anisotropic assemblies. The used simulation method and the developed algorithm allowed us to describe in detail the mutual arrangement of the patchy domains in the interlinked particles; however, in the situations where the number of patches was high, the possible combinations of the arrangement led to a wide distribution of the intermolecular mutual angles between the patches and the decomposition and detection of each link pattern became very tricky. Despite the technical difficulties, we were able to distinguish few patterns, such as patches aligned in a straight line, Y- and T-shaped links, which were repeatedly found in the assembled structures. The presence of these patterns which implicate multiple patches could be a first indication that the mikto-arm stars resembling the patchy particles may form interconnected network-like structures. We speculate that, at the higher weight fractions, the assemblies would be internally linked in a more complex way, similarly to the assemblies prepared here by the “assembly-first” approach. Interestingly, very similar patterns have been found during the crystallization process of triblock Janus colloids [97,98,99]. Triblock Janus colloids consist of colloidal particles with the two “poles” covered by patchy regions and thus resemble our mikto-arm stars with two patches aligned “in line”. More specifically, Eslami et al. showed that, by increasing the width of the patchy regions, the observed aggregates varied from strings through the Kagome lattice to the hexagonal phase [97]. The maximum width of the patchy region in Ref. [97] was 65 degrees, which is slightly smaller than our 90${}^{\circ }$ criterion for the patch recognition, but within the tested values of the algorithm versatility (see the Supplementary Materials).

## 5. Conclusions

Mikto-arm stars resembling patchy particles are a very recent addition to the family of nanosegregated particles. Here, we investigated an anisotropic self-assembly of mikto-arm stars consisting of 16 PEO and 16 PS arms in linear PB matrix. Internally interconnected domains composed of nanosegregated PEO arms contributed to the formation of links among spherical, mildly penetrated mikto-arm stars with a moderate number of patches. By varying the number of building blocks (stars) in the systems, we were able to describe in detail the mutual orientation of the patchy regions starting from a simple single-star and bimolecular structures, up to more complex, highly interlinked patterns.

Furthermore, we presented an algorithm based on the geometric alignment of the arms which was able to detect the segregated regions in the molecule. Due to the fact that the PEO arms are not constrained in the patchy region and freely rearrange, we observed a large variety of the arm and patch alignment. Three frequently appearing ways of a link formation were identified: patches aligned almost in a straight line and multi-domain Y-shape and T-shape patterns. A further investigation of bigger samples prepared by diverse preparation protocols is necessary to obtain enough information to complete the picture and be able to build a predictive tool which would facilitate the usage of the mikto-arm stars as soft patchy particles in nanosegregated materials. Nevertheless, the results obtained in our study confirm the potential of the mikto-arm stars to become a very versatile building block with penetrable and adjustable segregated domains.

## Figures and Tables

**Table 1:** Characteristics of the systems. *Rg* denotes the star radius of gyration, *Re* the size of the arm center-to-end vector, and *Rg_a_* the arm radius of gyration.

System	Rg [nm]	Arms	Re [nm]	${\mathrm{Rg}}_{a}$ [nm]
1s/PB	3.32 ± 0.06	PEO	3.97 ± 0.80	2.75 ± 0.11
		PS	4.48 ± 1.51	3.50 ± 0.08
2s/PB	3.37 ± 0.04	PEO	3.81 ± 0.67	2.52 ± 0.07
		PS	4.98 ± 0.64	3.50 ± 0.06
3s/PB	3.40 ± 0.04	PEO	3.78 ± 0.70	2.68 ± 0.07
		PS	4.55 ± 0.48	3.50 ± 0.05
4s/PB	3.43 ± 0.03	PEO	4.10 ± 0.53	3.00 ± 0.07
		PS	4.39 ± 0.42	3.56 ± 0.04
6s/PB	3.38 ± 0.03	PEO	4.07 ± 0.28	2.91 ± 0.04
		PS	4.43 ± 0.25	3.52 ± 0.03
8s/PB	3.42 ± 0.03	PEO	3.86 ± 0.32	2.98 ± 0.03
		PS	5.03 ± 0.29	3.56 ± 0.03

## Data Availability

The data presented in this study are available on request from the corresponding author.

## Supplementary Materials

The following are available online at https://www.mdpi.com/article/10.3390/polym13071114/s1, Figure S1: Prolateness parameter, Figure S2: Asphericity of the PS arms, Figure S3: Radial distribution functions of the distances among the centers of mass of the arms, Figure S4: Local number density for matrix monomers. Figures S5 and S6: Description and a test case for the algorithm detecting patches. Figures S7 and S8: Additional data for the systems prepared by the “assembly-first” method.

## Abbreviations

The following abbreviations are used in this manuscript:

PEO	poly(ethylene oxide)
PS	polystyrene
PB	polybutadiene
