# Supplementary material for: Mikto-Arm Stars as Soft-Patchy Particles: From Building Blocks to Mesoscopic Structures
Source: Polymers (Basel). 2021 Apr 1;13(7):1114. doi: 10.3390/polym13071114 (PMC8037958; doi:10.3390/polym13071114)
Supplement: Supplementary file 1 [file polymers-13-01114-s001.pdf]

SUPPLEMENTARY MATERIALS: Mikto-Arm Stars as Soft-Patchy Particles: From Building Blocks to Mesoscopic Structures

Petra Bačová,[1,2,*] Dimitris G. Mintis,[2] Eirini Gkolfi,[1,3] and Vagelis Harmandaris[1,3,2]

[1] *Institute of Applied and Computational Mathematics (IACM), Foundation for Research and Technology Hellas (FORTH), GR-70013 Heraklion, Crete, Greece*

[2] *Computation-based Science and Technology Research Center, The Cyprus Institute, 20 Constantinou Kavafi Str., Nicosia 2121, Cyprus*

[3] *Department of Mathematics and Applied Mathematics, University of Crete, GR-71409 Heraklion, Crete, Greece*

Prolateness parameter

The prolateness parameter was calculated as follows:

$$p = \frac{(2\lambda_1 - \lambda_2 - \lambda_3)(2\lambda_2 - \lambda_1 - \lambda_3)(2\lambda_3 - \lambda_1 - \lambda_2)}{2(\lambda_1^2 + \lambda_2^2 + \lambda_3^2 - \lambda_1\lambda_2 - \lambda_1\lambda_3 - \lambda_2\lambda_3)^{3/2}} \quad (1)$$

A perfectly oblate object is characterized by $p = -1$, a perfectly prolate object by $p = 1$.

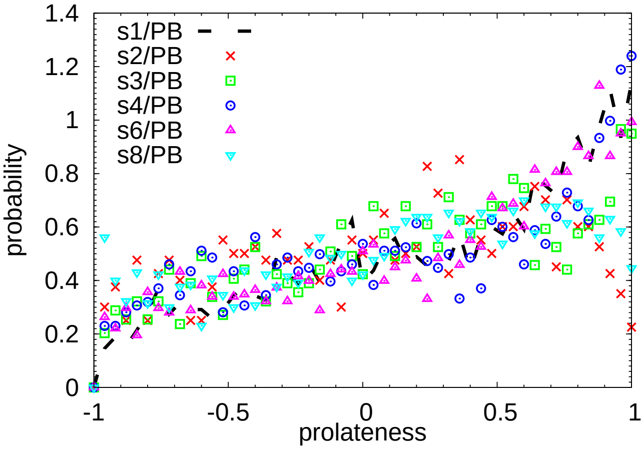

Figure S1: The probability distribution function of the prolateness of the star molecules.

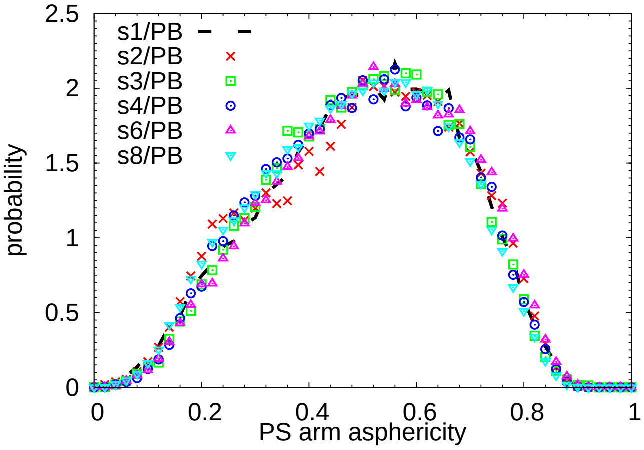

Figure S2: The probability distribution function of the asphericity of the PS arm.

*Electronic address: pbacova@iacm.forth.gr

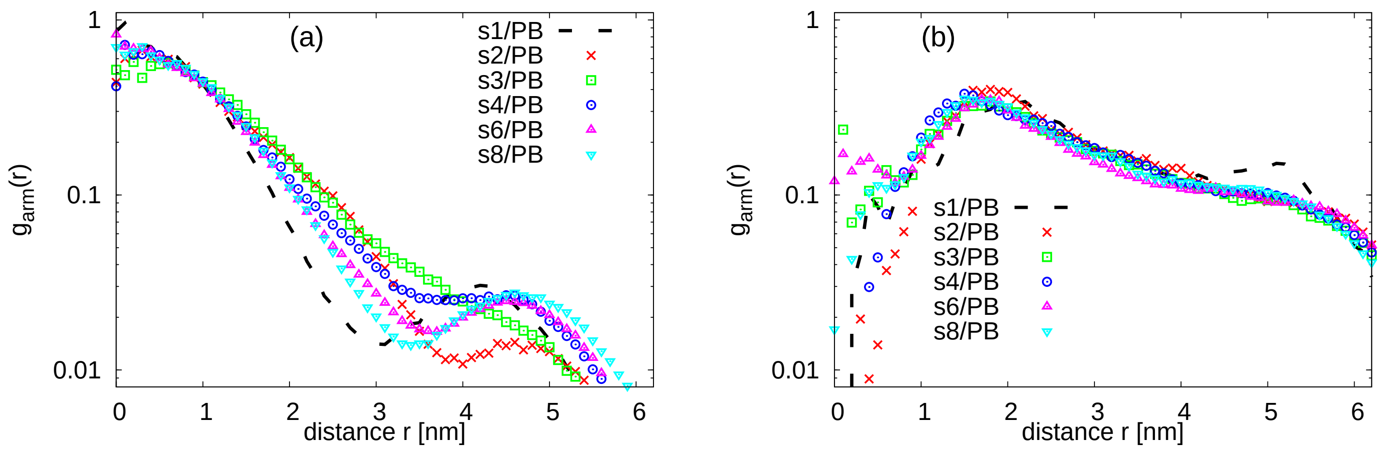

Figure S3: The intramolecular space arrangement of the arms: the radial distribution function of the distances among the centers of mass of the (a) PEO and (b) PS arms.

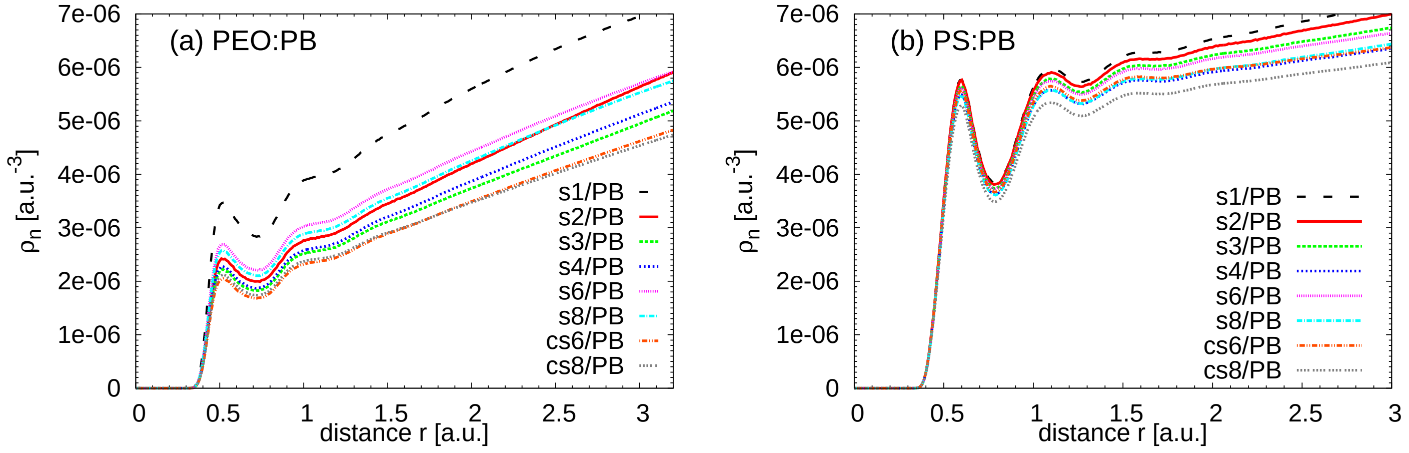

Figure S4: Local number density ρ_n for monomers of the PB matrix as a function of the distance r from (a) the PEO and (b) PS monomers placed on the star arms. The lower values of ρ_n mean that the star component can more efficiently avoid the interaction with monomers of the PB matrix.

Algorithm for detection of patchy domains

To allocate arms into segregated regions, first we define the vector from the central atom of the dendritic core to the center of mass of the arm. Then we select one arm, which by definition belongs to the first patch and calculate the angle $\theta = \arccos\left(\frac{\mathbf{v}_i \cdot \mathbf{v}_{i+1}}{\|\mathbf{v}_i\| \|\mathbf{v}_{i+1}\|}\right)$ between the selected vector and the one corresponding to the consecutive arm, i.e., vectors belonging to the arms attached to the dendritic core next to each other (see Fig. S5). If the mutual angle is $\theta < \theta_{max}$, these two arms are allocated into one patch. If $\theta \geq \theta_{max}$, the $i+1$ arm is included in the next patch and so on. Every time we move to the next patch we also examine a possible merging of previous patches by calculating the angle between the vector belonging to the newly assigned patch and the the vector belonging to the previously examined patch (see the magenta arc in Fig. S5). To test the versatility of the algorithm, we vary the angle θ_{max} in the range 45-115 °. The results for s4/PB are shown in Fig. S6, analogous results for the remaining systems were found. Within the error bars, we got the same results for θ_{max}=60, 75 and 90°. Results obtained for the two limiting cases θ_{max}=45° and θ_{max}=115° deviate the most and also overestimate and underestimate, respectively, the number of patches found by the visual inspection of the simulation snapshots.

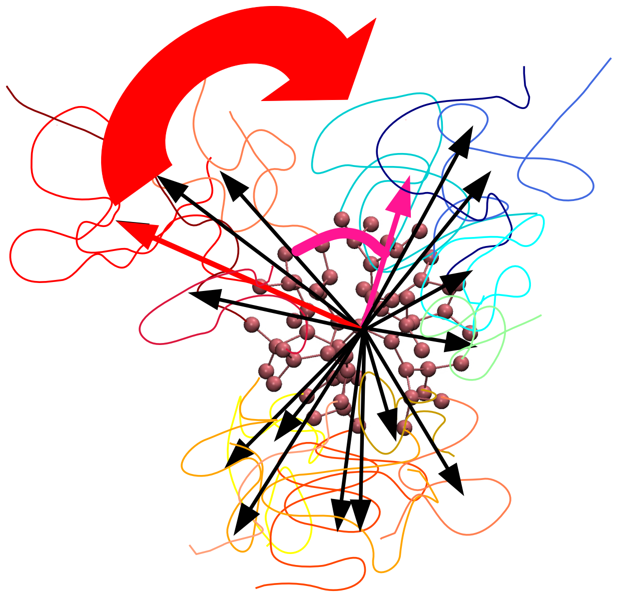

Figure S5: Schematic illustration of the algorithm used for the detection of patches. The arms arranged in the same segregated region are drawn by lines with the shades of the same color. The arms are attached schematically into a snapshot of the dendritic core. The big red arrow denotes the order in which the arms are checked in the algorithm. The red arrow illustrates the first vector $\mathbf{v_i}$, which is by defalut labelled as the first patch. The magenta arrow is the first one in the second patch, the magenta arc depicts the angle θ.

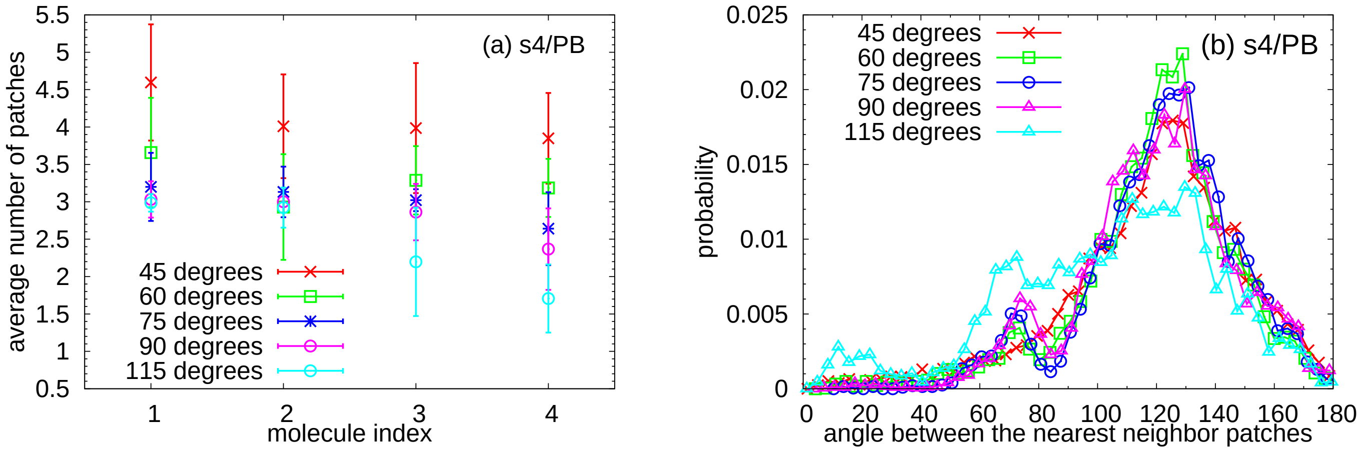

Figure S6: The effect of the selected angle in the criterion for the detection of the internal patchy regions on the results obtained from the detection algorithm. (a) The average number of patches per star in s4/PB system as a function of the star index. (b) The distribution of the intermolecular angles between the nearest neighbor patches.

Comparison of the two preparation methods

The initial configurations of simulated systems with 6 and 8 stars were prepared by 2 different protocols: "dispersed-first" method, starting from a random dispersion of the stars and and "assembly-first" protocol, starting from a configuration where stars were distributed regularly and assembled in vacuum. The systems prepared by the first method are labelled as s6/PB and s8/PB, those prepared by the second method as cs6/PB and cs8/PB.

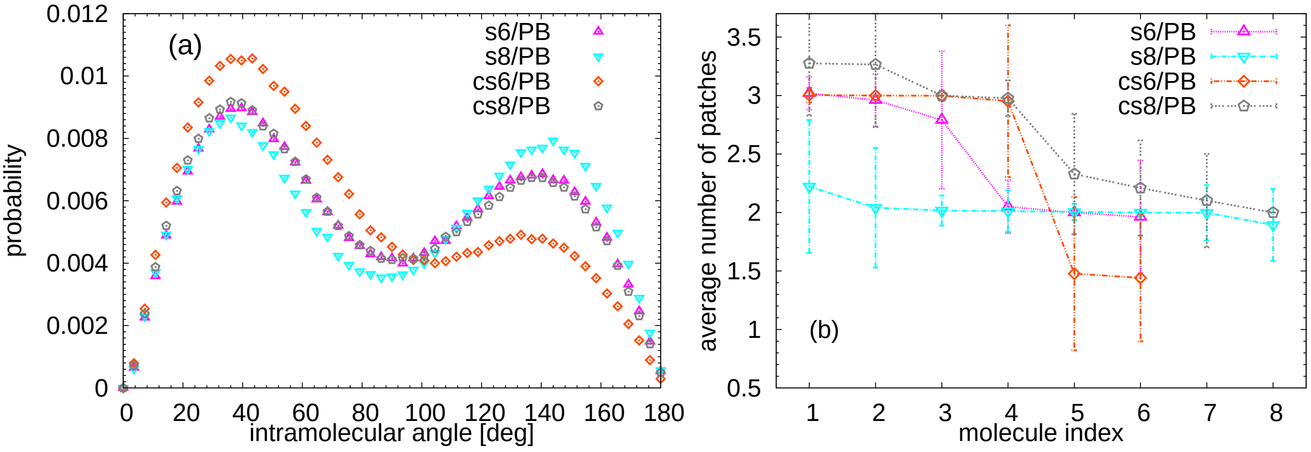

Figure S7: The intramolecular properties of the stars prepared by "assembly-first" method in comparison to those prepared by "dispersed-first" approach. (a) The distributions of the angles among the end-to-end vectors of the PEO arms averaged over all the stars in the system. (b) The average number of patches per star plotted as an index of the molecule in the system. Stars were sorted and labeled in descending order of the number of patches. The lines in (b) are guide for the eye.

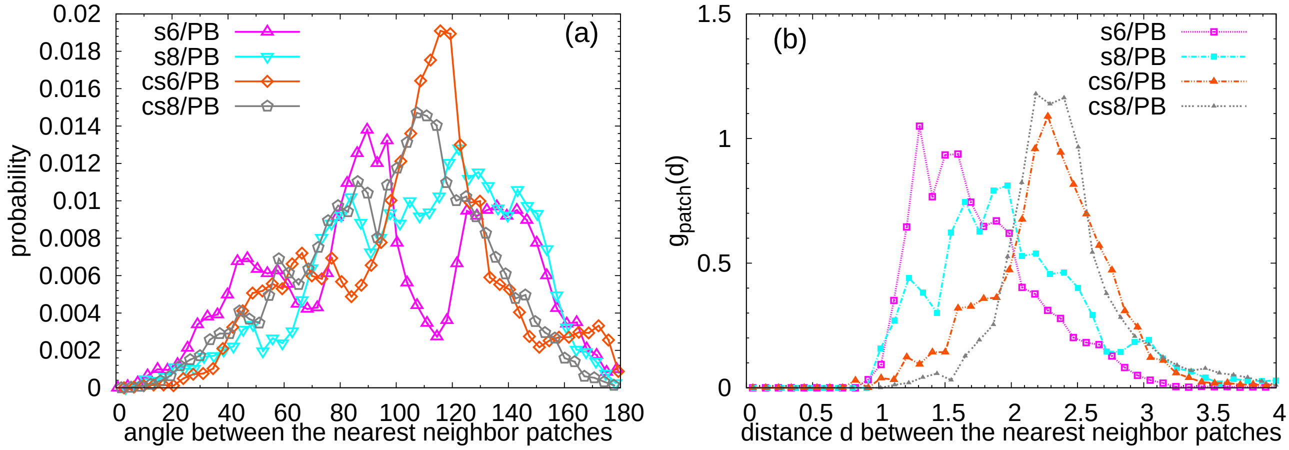

Figure S8: (a) The probability distribution function of the intermolecular angles between the nearest neighbor patches and (b) the radial distribution function of the distances between the centers of mass of these patchy regions in s6 and s8/PB systems in compariosn to the cs6 and cs8/PB analogues.
